# The Arm Movement Detection (AMD) test: a fast robotic test of proprioceptive acuity in the arm

**DOI:** 10.1186/s12984-017-0269-3

**Published:** 2017-06-28

**Authors:** Leigh Ann Mrotek, Maria Bengtson, Tina Stoeckmann, Lior Botzer, Claude P. Ghez, John McGuire, Robert A. Scheidt

**Affiliations:** 1 Marquette University, Biomedical Engineering 1515 W. Wisconsin Ave, Milwaukee, WI 53233 USA; 2 Marquette University, Physical Therapy P.O. Box 1881, Milwaukee, WI 53201-1881 USA; 3 Columbia University, Neuroscience Kolb Annex, 1051 Riverside Drive, New York, NY 10032 USA; 4 Medical College of Wisconsin, Physical Medicine and Rehabilitation 9200 W. Wisconsin Ave, Milwaukee, WI 53226 USA; 5 Northwestern University Feinberg School of Medicine, Physical Medicine and Rehabilitation 710 North Lake Shore Drive #1022, Chicago, IL 60611 USA; 6 University of Wisconsin Oshkosh, Department of Kinesiology, 800 Algoma Boulevard, Oshkosh, WI 54901-8630 USA

**Keywords:** Proprioception, Threshold, Uncertainty, Aging, Stroke, Force, Displacement

## Abstract

**Background:**

We examined the validity and reliability of a short robotic test of upper limb proprioception, the Arm Movement Detection (AMD) test, which yields a ratio-scaled, objective outcome measure to be used for evaluating the impact of sensory deficits on impairments of motor control, motor adaptation and functional recovery in stroke survivors.

**Methods:**

Subjects grasped the handle of a horizontal planar robot, with their arm and the robot hidden from view. The robot applied graded force perturbations, which produced small displacements of the handle. The AMD test required subjects to respond verbally to queries regarding whether or not they detected arm motions. Each participant completed ten, 60s trials; in five of the trials, force perturbations were increased in small increments until the participant detected motion while in the others, perturbations were decreased until the participant could no longer detect motion. The mean and standard deviation of the 10 movement detection thresholds were used to compute a Proprioceptive Acuity Score (PAS). Based on the sensitivity and consistency of the estimated thresholds, the PAS quantifies the likelihood that proprioception is intact. Lower PAS scores correspond to higher proprioceptive acuity. Thirty-nine participants completed the AMD test, consisting of 25 neurologically intact control participants (NIC), seven survivors of stroke with intact proprioception in the more affected limb (HSS+P), and seven survivors of stroke with impaired or absent proprioception in the more affected limb (HSS-P).

**Results:**

Significant group differences were found, with the NIC and HSS+P groups having lower (i.e., better) PAS scores than the HSS-P group. A subset of the participants completed the AMD test multiple times and the AMD test was found to be reliable across repetitions.

**Conclusions:**

The AMD test required less than 15 min to complete and provided an objective, ratio-scaled measure of proprioceptive acuity in the upper limb. In the future, this test could be utilized to evaluate the contributions of sensory deficits to motor recovery following stroke.

**Electronic supplementary material:**

The online version of this article (doi:10.1186/s12984-017-0269-3) contains supplementary material, which is available to authorized users.

## Background

Limb proprioception is the complex sensation of limb posture and movement [3] derived from multiple sensory receptors that signal the physical state of the limb (e.g., information about the configuration of the joints and changes in joint configuration). These receptors include mechanoreceptors in muscles, tendons, joints, and areas of skin overlying the joints (c.f., [47]). Proprioceptors provide critical state information used for planning of multijoint movements [28, 30, 49, 50, 57, 58] and for perceiving the sensory consequences of those actions [32, 59, 60]. Proprioception also contributes importantly to the ongoing, closed-loop, feedback control of stabilized limb postures and goal-directed movements [17, 21, 29, 63] as well as to the learning of coordinated skills such as handwriting and feeding oneself [46, 48, 68]. Several pathological conditions lead to progressive or sudden loss of proprioceptive sensation, thereby impairing ability to perceive orientation, velocity, and force production in a limb. After stroke, for example, the loss of upper extremity proprioceptive sensation can degrade sensorimotor control of actions vital to an independent life style [13, 40, 70], including reaching, stabilizing the limb, and manipulating objects [5, 53, 65, 69].

To develop a better understanding of the causal relationship between proprioceptive loss, motor disability, and the capacity for recovery of sensorimotor function after neuromotor injury, it is necessary to quantify proprioceptive integrity and its impact on motor planning and control. Currently there are several quick clinical tests of proprioception that yield subjective assessments of proprioceptive integrity on an ordinal scale. One of these is the “Up or down?” test [20, 23]. Here, the participant closes his or her eyes while a clinician moves the distal limb segment of the tested joint up and down several times with respect to horizontal, taking care to avoid proximal pressure and gravitational cues related to the movement. When the clinician stops moving the joint, the participant is prompted to verbally indicate joint orientation. Multiple repetitions are performed at each joint. If responses are brisk and accurate (i.e. the participant makes no errors), proprioception is rated “intact”; if the participant is unable to respond with confidence (i.e., they make 1 error), proprioception is rated “impaired”; if the participant is unable to determine position reliably (2 or more errors), proprioception is rated “absent”. A limitation of this test is the ordinal nature of participant responses and clinician assessments, both of which limit the sensitivity and specificity of the test. A second limitation is the ceiling effect observed in neurologically intact individuals, which precludes sensitivity to subtle changes in sensory acuity such as those that occur as a natural consequence of aging [1, 24]. Moreover, due to the difficulty in eliminating secondary cues related to the imposed motions - especially at more proximal joints - results of the “up or down?” test can be easily confounded. In another common clinical test, the position matching test, the unseen thumb of one hand is moved passively to several reference locations and the participant’s task is to actively reproduce the position with the other limb. Although matching tests are well-suited for assessing the integrity of the entire neuromuscular arc spanning both limbs, they lack specificity because test results are sensitive to proprioceptive deficits in either limb as well as deficits in the central integration of that sensory information, thus confounding proprioceptive deficits in the arm under evaluation with sensory and motor deficits that can frequently occur in the matching limb [39, 43, 62]. Thus, for researchers interested in elucidating the impact of proprioceptive impairment on functional recovery post-stroke, current clinical tests suffer from several limitations. These include poor reliability, limited sensitivity and specificity, and from the fact that they yield results on an ordinal scale, which does not discriminate subtle differences in proprioceptive acuity, rather than a continuous, ratio scale which can account for these differences [18, 27, 36, 37].

Recognizing these limitations, several groups have designed standardized tests [11, 12, 38, 67] and automated procedures [22, 31, 44, 61] that quantify somatosensory deficits by assessing a person’s ability to identify the static posture of an unseen limb [12] or to indicate when a passively moved limb has changed position [44]. For example, Carey and colleagues have developed a position sense apparatus and test procedure wherein an examiner moves the unseen wrist to a predefined position, and the participant indicates perceived wrist angle by aligning a goniometric pointer with an imagined line linking the wrist center of rotation and the index finger [12]. Although this approach avoids confounds associated with imitating or matching tasks that involve both limbs, Carey’s test does not evaluate the participant’s ability to sense limb motion, which is also important for the control of multijoint movement [29, 51]. By contrast, Niessen and colleagues developed a test that requires participants to indicate when they detect motion in a slowly moving arm or when the moving limb’s position matches a previously presented position [44]. Building on that work, Simo and colleagues developed a test procedure using a two-alternative forced choice approach from sensory psychophysics (the “method of constant stimuli”) to quantify stroke-related changes in detection thresholds for arm movements elicited by robotic force and displacement perturbations [55]. While that approach yielded an accurate, ratio scaled test of proprioceptive integrity within the tested limb, it required at least 45 min to implement, thus limiting its utility for most purposes beyond research.

We therefore set out to improve on the automated approaches established by Niessen et al. [44] and Simo et al. [55] to develop a quick, quantitative, robotic test of whole-arm proprioception that gives objective results on a ratio scale. We refer to this new robotic test as the Arm Movement Detection (AMD) test, reflecting the specific task posed to the examinee. We focus our study here on stroke survivors because stroke is a brain injury that commonly compromises proprioceptive sensation; almost 60% of stroke survivors experience impaired limb position sense in their contralesional arm [9] which can negatively impact activities of daily living [10, 19, 66]. We report AMD test results obtained from stroke survivors in comparison to those obtained from neurologically intact control participants that spanned a wide range of ages. The robotic test we describe requires an assessment time of less than 15 min and uses small force perturbations and the motions they produce to obtain an objective, accurate, reliable, and ratio-scaled measure of proprioceptive integrity. Portions of this work have previously appeared in abstract form [4, 42].

## Methods

Thirty-nine volunteers provided informed consent to participate in institutionally reviewed and approved experimental procedures at Marquette University. Fourteen participants were hemiparetic survivors of stroke (HSS) presenting with contralesional hemiparesis. Inclusion criteria for HSS participants included: sufficient shoulder and elbow range of motion to sit with the arm in the plane of the robot for up to one hour. Exclusion criteria included: the inability to follow two-step instructions (assessed during participant screening); fixed contracture or a history of tendon transfer in the involved limb; subjects with a bleeding disorder; and subjects diagnosed with myasthenia gravis, amyotrophic lateral sclerosis or any other disease that might interfere with neuromuscular function. We did not exclude participants based on recent or concurrent botulinum neurotoxin therapy in the involved limb, and four participants had received botulinum neurotoxin injections within three months of participation.

HSS participants completed the experiment with the more affected arm (Table 1). Seven participants had impaired or absent proprioception in the more affected upper extremity, as determined by the “up or down?” test [HSS-P; age 57 ± 10 year (mean ± SD, here and elsewhere)]. The remaining seven HSS participants presented with intact proprioception in the more affected limb (HSS + P; age 63 ± 6 year). Twenty-five neurologically intact control participants (NIC) between the ages of 18 and 87 (47 ± 22 year) also completed the experiment. None of the NIC participants had known neurological or muscular disorders. NIC participants completed the experiment with the dominant (preferred) arm; a subset of this group (*n* = 16) also agreed to complete the experiment with the non-preferred arm. Another subset (*n* = 7) was available to return on two separate, subsequent days in order to repeat the experiment with the preferred arm.

**Table 1:** Hemiparetic stroke survivor demographic data

ID	Status	Handle	Age	Sex	Years post-stroke	Lesion	Test side	MoCA	FM_M_	FM_prop._	FM_LT_	MAS^c^	PAS
101	HSS-P	Cylinder	57	M	12	I/SC	L	27	27	2_S_, 1_E_, 0_W_, 0_T_	2	0.38	0.49 ± 0.22
107	HSS-P	Cylinder	61	M	12	I/C	R	10^b^	27	1_S_, 1_E_, 0_W_, 0_T_	2	1.0	1.30 ± 0.44
108^a^	HSS-P	Plane	61	M	9	I/C	L	28	9	2_S_, 2_E_, 0_W_, 1_T_	2	1.13	0.48 ± 0.39
106	HSS-P	Cylinder	64	F	24	*/*	R	14^b^	45	2_S_, 1_E_, 1_W_, 0_T_	1	0.75	0.95 ± 0.43
105	HSS-P	Sphere	61	M	9	I/SC	R	25	66	1_S_, 1_E_, 1_W_, 1_T_	2	0	0.75 ± 0.06
112^a^	HSS-P	Cylinder	34	M	6	H/*	L	27	21	2_S_, 2_E_, 1_W_, 0_T_	1	1.63	0.85 ± 0.21
113	HSS-P	Cylinder	63	F	10	*/*	L	22	37	2_S_, 2_E_, 1_W_, 0_T_	2	1.38	0.33 ± 0.11
102	HSS + P	Cylinder	59	M	7	I/SC	R	26^b^	20	2_S_, 2_E_, 2_W_, 2_T_	4	1.5	0.51 ± 0.05
104	HSS + P	Cylinder	52	M	13	*/*	R	23^b^	21	2_S_, 2_E_, 2_W_, 2_T_	2	1.38	0.45 ± 0.11
103^a^	HSS + P	Cylinder	64	F	29	*/*	L	26	30	2_S_, 2_E_, 2_W_, 2_T_	4	2.75	0.28 ± 0.07
110	HSS + P	Sphere	62	M	7	I/SC	L	23	41	2_S_, 2_E_, 2_W_, 2_T_	4	1.63	0.46 ± 0.13
111	HSS + P	Sphere	69	F	35	H/B	R	25	23	2_S_, 2_E_, 2_W_, 2_T_	4	0.75	0.43 ± 0.06
114	HSS + P	Sphere	64	M	7	I/SC	L	24	66	2_S_, 2_E_, 2_W_, 2_T_	4	0	0.35 ± 0.18
115	HSS + P	Cylinder	70	F	13	H/*	L	22	32	2_S_, 2_E_, 2_W_, 2_T_	4	0.375	0.42 ± 0.08

*Abbreviations: ID* patient identifier, *HSS-P* hemiparetic stroke survivor with impaired proprioception, *HSS + P* hemiparetic stroke survivor with intact proprioception, *M* male, *F* female, Lesion{type/location} - *I* ischemic, *H* hemorrhagic, *C* cortical, *SC* subcortical, *B* brainstem, Test Side - *L* left, *R* right, *MoCA* montreal cognitive assessment score, *FM*_*M*_ motor portion of the Fugl-Meyer Assessment, *FM*_*PROP*_ proprioceptive component of the sensory portion of the Fugl-Meyer Assessment, *S* shoulder, *E* elbow, *W* wrist, *T* thumb, *FM*_*LT*_ light touch component of the sensory portion of the Fugl-Meyer Assessment, *C-13* the chedoke McMaster Arm and Hand Inventory - 13-element version, *MAS* modified ashworth scale, *PAS* proprioceptive acuity score (see Methods for computational details)

*Information unavailable

^a^Participants received botulinum neurotoxin treatment within three months prior to testing

^b^Participant has expressive aphasia

^c^MAS score was computed by averaging the individual MAS scores for Elbow flexion & extension and shoulder abduction & adduction

All HSS participated in an additional evaluation session in which a licensed physical therapist assessed sensorimotor function and impairment with the participant seated in an armless chair (Table 1). Clinical assessments included the sensory and motor portions of the Upper Extremity Fugl-Meyer assessment (UEFM; [26]) and the modified Ashworth scale (MAS; [7]) assessing abnormal muscle tone at the shoulder, elbow and wrist. The sensory portion of the UEFM evaluates proprioceptive discrimination at the shoulder, elbow, wrist, and metacarpophalangeal articulations using the “*up or down?*” test [20, 23] described earlier.

### Experimental procedures

**Fig. 1:**
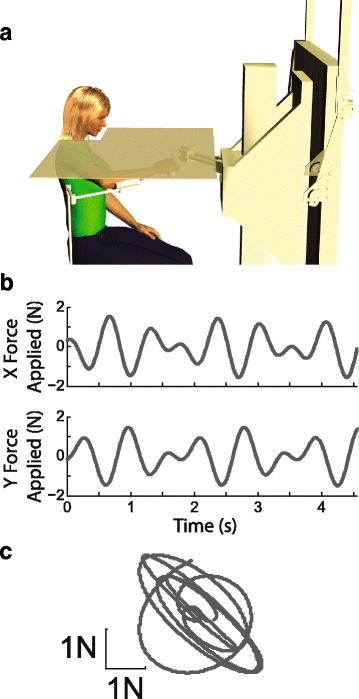
Experiment Setup. **a** Participants sat in a high backed chair with shoulder, arm and hand supported and hidden from view. The hand was mechanically coupled to the handle of a horizontal planar robot. Participants were instructed to relax the arm at all times during the experiment. The robot perturbed the hand with forces that followed a complex sum-of-sinusoids pattern during each 60 s trial. The experimenter adjusted force magnitude based on the participant’s verbal responses to questions designed to identify the psychophysical threshold of movement detection. **b** Hand forces applied in the x-direction (*top*) and y-direction (*bottom*) for the first five cycles of the slowest component of the sum-of-sinusoids perturbation. **c** Corresponding XY forces applied during the same five cycles for a neurologically intact control participant

Participants were seated in a high-backed chair in front of a horizontal, planar, 2-joint robotic manipulator equipped with a handle which subjects were to grasp (Fig. 1a). This custom, low-inertia device was powered by two brushless DC torque motors (M-605-A Goldline; Kollmorgen, Inc. Northampton, MA), and it allowed movements only within the horizontal plane. A 16-bit data acquisition board (PCI-6031E DAQ; National Instruments Inc., Austin, TX) sampled analog force from a load cell (85M35A-I40-A-200 N12; JR3 Inc., Woodland, CA) mounted under the handle. Handle location was resolved within 0.038 mm using joint angular position data from two 17-bit encoders (A25SB17P180C06E1CN; Gurley Instruments Inc., Troy, NY). Data collection and control were performed at 1000 sample/s. Additional details of the device have been described previously [52].

**Fig. 2:**
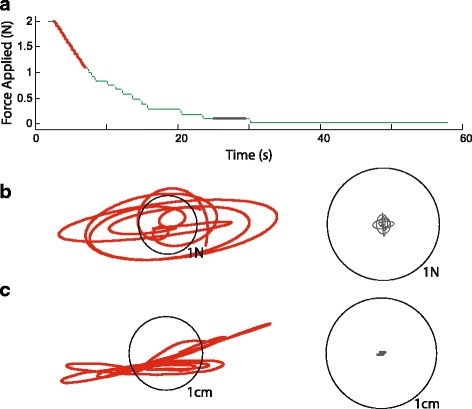
Evolution of hand forces during a typical descending trial, as performed by a neurologically intact control participant. **a** Force magnitude as a function of time for a single trial. **b** and **c** A comparison of hand forces and motions from the initial (*red*) and later (*grey*) parts of the trial. The thin black circular line represents a scale of 1 N or 1 cm (respectively) for each trace

**Fig. 3:**
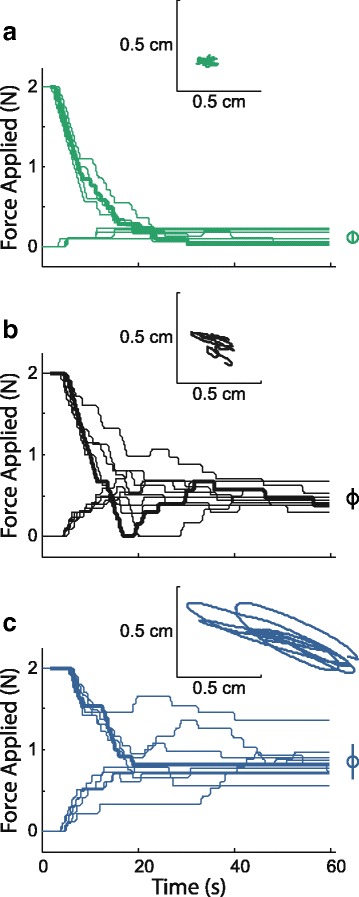
Hand force time series for selected participants. **a** Neurologically intact control participant (NIC). The bolded trial in the NIC plot is the same as shown in Fig. 2. **b** Hemiparetic Stroke Survivor with intact proprioception (HSS + P). **c** Hemiparetic Stroke Survivor with impaired proprioception (HSS-P). Each panel depicts the magnitude of force [N] applied to the participant’s hand as a function of time in each 60-s trial. The across-trials mean and standard deviation values of the final force magnitudes are graphically depicted on the far right side of each panel. Insets: Hand path displacements during the last five full cycles of the slowest component of the force perturbation, as measured during the bolded trial in the main panel

**Fig. 4:**
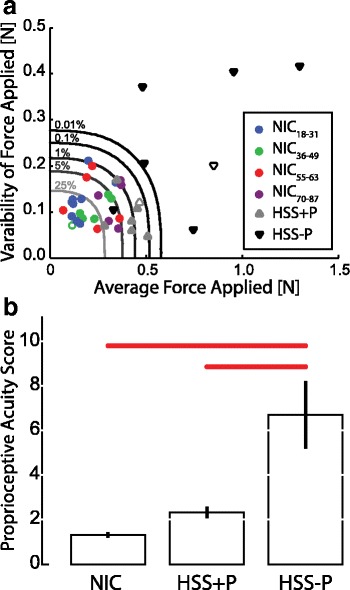
Primary experimental findings. **a** Plot showing the variability (i.e., standard deviation *V*; units: [N]) of the threshold plotted vs. mean threshold (*TH*; units: [N]). neurologically intact control group (NIC); hemiparetic stroke survivors with intact proprioception (HSS+P); hemiparetic stroke survivors without intact proprioception (HSS-P). The NIC participants are represented by circles and subdivided into four groups based on age: *blue circles* are younger participants (*n* = 9; age range: 18–31 year; 24 ± 5 year), *green circles* are the middle-young participants (*n* = 6; age range 36–49 year; 42 ± 5 year), *red circles* are the middle-old participants (*n* = 5; age range 55–63 year; 60 ± 3 year), and *purple circles* are the older participants (*n* = 5; age range 70–87 year; 80 ± 7 year). The HSS+P group is represented by gray triangles pointing up and the HSS-P group is represented by *black triangles* pointing down. Each group has one “hollow” marker denoting that those participants’ data are represented in Fig. 3. Iso-likelihood lines show several difference confidence bounds for likelihood of normal proprioceptive sensation. The darkest line is the 0.01% iso-likelihood line (chance of missing a participant who has normal proprioception), the next line is the 0.1% then 1.0%, 5%, and 25%. **b** Group average Proprioceptive Acuity Score (PAS) with group error bars (± 1 SEM). Scores for the NIC, HSS+P and HSS-P groups. PAS is a measure of the distance of a point from “normal” performance, as defined using NIC performance data (see Methods). *Red bars* indicate that the members of the HSS-P group had significantly greater PAS than the members of the other two groups

**Fig. 5:**
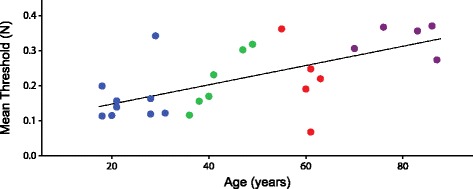
Mean detection threshold (*TH*) vs. age for the NIC participants. There is a slight but significant increase in the threshold with increasing age (*TH* = 0.090 + 0.003*Age). *Blue circles*: younger; *Green*: middle-young; *Red*: middle-old; *Purple*: older

The experimental task did not require participants to have functional movement of the arm and/or hand and fingers. We did however require that participants have sufficient passive range of motion at the shoulder such that the arm could be supported comfortably against gravity at the level of the handle (75° to 90° abduction; ≈60° horizontal flexion) using a light-weight, chair-mounted support or ceiling-mounted sling (Fig. 1a). We used three different handle configurations {spherical, vertical cylinder, horizontal plate} and supplemental Velcro® straps to assure safety, comfort and the most secure mechanical coupling of the arm to the robot despite varying degrees of paresis and spasticity (Table 1). If survivors could pronate the forearm and we could conform and/or strap the hand to the sphere, then that handle was used. If not, we then attempted to use the vertical cylinder. If the hand could not be secured to the cylinder with Velcro® wrap, we instead used a wrist splint and Velcro® to fix the survivor’s wrist to the horizontal plate. An opaque screen above the plane of hand motion occluded view of shoulder, arm and robot. Participants wore noise-canceling headphones to diminish slight sounds produced by the robot’s motors. Participants were instructed to relax the supported arm at all times during experimental testing.

During the experiment the robot applied small horizontal planar forces to the hand, which induced small motions of the arm that occurred mainly at the shoulder and elbow joints. We determined the smallest magnitude of hand force perturbations - and the resulting motions - that participants could detect reliably. We did so by characterizing each participant’s ability to detect small planar perturbations of the handle during a series of ten interactive, “method-of-adjustment” trials at a single comfortable location in the center of the arm’s reachable workspace. We programmed the robot to generate hand forces that were composed of separate sum-of-sinusoids in the “X” (1.75 Hz and 1.2 Hz) and “Y” (1.65 Hz and 1.1 Hz) directions (cf. Fig. 1b). During odd numbered trials (*descending staircase* trials), the robot initially perturbed the hand with a maximal force of 4 N peak to peak; all participants verbally indicated that they could feel the motion induced by this perturbation. Participants then had 60 s within which they responded to the repeated question: "Do you feel your arm moving?" The experimenter decreased the perturbation magnitude after each affirmative response. This process repeated until the participant felt the arm cease moving. On even numbered trials (*ascending staircase* trials), the robot arm initially applied no forces and participants were to respond to the verbal prompt. Based on participant responses, the experimenter adjusted the perturbation magnitude iteratively until the participant could just begin to feel the arm moving. For both trial types, each change in response (yes to no or vice versa) caused the experimenter to change the sign of force increment/decrement, thus refining the participant’s threshold estimate for that trial. In this way, each 60-s trial yielded an estimate of the participant’s psychophysical threshold for arm movement detection.

### Data analysis and statistical hypothesis testing

We calculated two primary measures of performance based on each individual’s responses: *Mean arm movement detection threshold* (*TH*) and the *variability (*i.e.*, standard deviation) of the ten assessments* (*V*). We also computed the *magnitude of X-Y hand motion* induced by robotic forces at the participant’s detection threshold by calculating hand path length during a time window that captures the last complete cycle of the slowest component of the applied force perturbation (i.e., within the last 910 ms of each trial). We extended the analysis described in Simo et al. [55] by combining *TH* and *V* to calculate a final outcome measure, the *Proprioceptive Acuity Score (PAS)*, which provides a measure of likelihood that proprioception is intact.

For each location within the {*TH, V*} plane, we computed the likelihood of intact proprioception as the product of the *TH* and *V* cumulative likelihood functions derived from normal distributions fit to the NIC group data. We then computed a composite performance measure, the PAS, as the standardized distance [34] between each individual’s data pair and the origin of the {*TH, V*} plane, thus allowing us to represent each individual’s {*TH, V*} data point as a likelihood estimate (i.e. a bivariate *Z*-score; Eq. (1).

1 $$ {PAS}_i=\sqrt{\left[{\left(\frac{TH_i-{TH}_{NIC}}{\sigma_{TH_{NIC}}}\right)}^2+{\left(\frac{V_i-{V}_{NIC}}{\sigma_{V_{NIC}}}\right)}^2\right]} $$

To calculate PAS for each individual participant (i.e., subscript *i*), we first subtracted the corresponding NIC population mean from his or her {*TH*_*i*_ *, V*_*i*_} data pair, then standardized the resulting values by dividing each component by the standard deviation values derived from the NIC participant *TH* and *V* distributions. We defined PAS as the Euclidean magnitude of the resulting vector.

We used general linear mixed model ANOVA with Scheffe’ post hoc analyses to test the hypothesis that PAS values vary across participant groups {NIC, HSS+P, HSS-P}, the hand under test {preferred, non-preferred; repeated measures}, and test repetition number {1, 2, 3; repeated measures}. We used linear regression to confirm the finding [55] that arm movement detection thresholds identified using the AMD test correlate strongly with the amount of limb motion observed at threshold. Finally, we used linear regression analysis to test the secondary hypothesis that within the NIC participant group, proprioceptive acuity degrades with aging [1, 24]. Statistical testing was performed in the SPSS computing environment [33]. Effects were considered significant using a family-wise error rate of α = 0.05.

## Results

All participants remained alert throughout the experimental session, and all readily understood the experimental instructions. All participants, even those presenting with expressive aphasia (Table 1), were able to quickly and effectively respond verbally to the test question. Thus, the AMD test was easily administered to a wide variety of participants. Across all participants, the total duration of the AMD test assessment - including brief rest intervals between trials - averaged less than 15 min (12:12 min ± 1:25 min).

Figure 2 depicts how robotic perturbations evolved during a representative descending staircase trial performed by a NIC participant, who demonstrated a low movement detection threshold. The overall force magnitude applied in the horizontal plane by the robot arm is shown in Fig. 2a. The robot’s sensors measured relatively large forces (Fig. 2b) and hand displacements (Fig. 2c) early in the trial (red highlighting). Across subjects, the hand displacements induced by the largest robotic perturbations corresponded to joint angular displacements of averaged approximately 4.9° ± 2.0° at the shoulder and 7.6° ± 3.5° at the elbow in the NIC participants, and 3.1° ± 1.7° at the shoulder and 6.0° ± 3.0° degrees at the elbow in the HSS participants. All members of all three participant groups reliably perceived arm motion with the strongest force perturbations. Near threshold (i.e., near the end of the trial), force and displacement magnitudes were measured to be much lower (gray highlighting). Across subjects, robotic perturbations at threshold induced displacements averaging 0.5° ± 0.5° at the shoulder and 0.6° ± 0.4° at the elbow in the NIC participants, and 1.0° ± 1.3° at the shoulder and 1.8° ± 2.6° at the elbow in the HSS participants.

We quantified the extent to which participants converged to similar force detection thresholds in both the descending and ascending trial type by quantifying the across-trial variability of threshold estimates for each participant. Figure 3 shows all trials for an arbitrarily selected participant from each of the three participant groups. Each line represents the time course of a single trial. The final force magnitude was used to compute each participant’s proprioceptive threshold. The mean and standard deviation of all ten trials performed by these participants are depicted graphically on the right side of each panel. The neurologically intact control participant (Fig. 3, top panel) demonstrated consistency in threshold determination by converging to the same threshold in both the descending and ascending trial types. For this NIC participant, the observed movement detection threshold was low, and variability around the threshold was small. Thus, this NIC participant had high sensitivity to arm movement and demonstrated consistent performance in the detection task. For the HSS+P participant (Fig. 3, middle panel), trials converged to a slightly higher threshold and demonstrated greater variability than that seen in the NIC participant. For the HSS-P participant (Fig. 3, bottom panel) the trials did not converge as well as for the other participants shown, and we observed a correspondingly larger standard deviation. As we show below, this general pattern of results was characteristic of our three participant groups.

The inset within each panel of Fig. 3 depicts the hand displacements recorded within the final five cycles of the bolded trial. As force threshold increased, the magnitude of hand displacements measured at threshold also increased. For the three participants depicted here, hand displacement at threshold averaged 0.063 cm per cycle (NIC), 0.085 cm per cycle (HSS + P) and 1.15 cm per cycle (HSS-P). For all tested limbs (*n* = 39) we observed a strong correlation between force magnitude and hand displacement at threshold (*r* = 0.734; *p* < 0.0005).

Normative confidence bounds for intact proprioception were computed using *TH* and *V* distributions estimated from the first AMD test assessments of the preferred hand in all control participants. The iso-likelihood curves in Fig. 4a depict loci of constant % likelihood of intact proprioception, which we use to visualize the distribution of detection thresholds within and across participant groups. Each iso-likelihood curve depicts the locus of {*TH, V*} data pairs corresponding to a given probability of misclassification, i.e., that an observation falling outside the convexity of that contour would have originated from a neurologically-intact participant. As expected, all the NIC participants (circles) fell within convexity of the 1% iso-likelihood contour.

Whereas four HSS+P participants also fell within the 1% iso-likelihood contour, thereby supporting their clinical assessment of intact proprioception, the remaining three HSS+P fell outside the 1% contour and were enclosed by the 0.1% contour, suggesting the presence of measurable degradation of proprioceptive acuity in these individuals. By contrast, six of the seven HSS-P participants with clinical assessments of impaired or absent proprioception fell outside the convexity of the 0.01% iso-likelihood contour, consistent with the presence of marked proprioceptive impairments in this group.

Note that both performance measures {*TH* and *V*} contribute information required to assess proprioceptive integrity in stroke survivors: although *TH* values for two of the HSS-P participants fell within the range of threshold values yielded by HSS+P participants, the corresponding HSS-P *V* values were outside the “normal” range for intact proprioception. Finally, one HSS-P participant suspected of proprioceptive impairment (based on the “up or down?” test) performed as well as many of the NIC participants on the AMD test. Given that the effect of one or two slow and/or erroneous responses would have little impact on the *TH* and *V* values provided by the AMD test but could yield a misclassification in the “up or down?” test, we believe it likely that this data point exposes a limitation in the specificity of the “up or down?” test.

The Proprioceptive Acuity Score (PAS) revealed marked differences in proprioceptive integrity between the groups (F_(2,36)_ = 27.665, *p* < 0.0005; Fig. 3b). The HSS-P participants had higher PAS values than the participants in the other two groups (Scheffe’ post hoc tests: *p* < 0.0005 in both cases), which did not differ from each other (*p* = 0.392). Regression analysis found that the functional relationship between PAS and hand path displacement at threshold (*r* = 0.742, *p* < 0.0005) supports the claim by Simo et al. [55] that force perturbations are well-suited for use in tests of limb proprioception pertaining to motion detection.

A subset of NIC participants performed the AMD test multiple times to allow comparison of test-retest reliability as well as laterality of the measure. Sixteen NIC participants repeated the AMD test a using the non-preferred hand to test laterality of the measure. We found no difference in PAS between the preferred and non-preferred hands (F_(1,30)_ = 0.024, *p* = 0.877). Seven NIC participants completed the test three times with the preferred hand in three separate sessions to test repeatability of the measure. No differences were found between test repeats (F_(2,18)_ = 0.650, *p* = 0.534). Taken together, the findings demonstrate that the AMD test has test-retest reliability and sufficient sensitivity to identify even subtle differences of proprioceptive sensation in stroke survivors.

Finally, within our cohort of NIC participants (*n* = 25) we examined the relationship between the mean threshold and age, and independently, the relationship between proprioceptive acuity (PAS) and age. We found a strong, positive correlation between mean threshold and age (*r* = 0.638, *p* = 0.001; Fig. 5). This result demonstrates that the AMD test is sensitive to subtle changes in proprioceptive acuity that naturally occur through the aging process [1, 24, 35, 41, 56]. Although the age related changes in proprioceptive acuity fall within the normal range, they are detected with this sensitive test but would not be detected with standard clinical tests of proprioception.

By contrast, we found no significant relationship between PAS and age within the NIC group (*r* = 0.224, *p* = 0.281). Although we have shown that the variability component of the PAS measure is helpful in characterizing proprioceptive impairment after stroke, variability in threshold assessment is evidently *not* informative of the proprioceptive changes that occur due to aging, masking a strong relationship between age and mean detection threshold. Taken together, our results reveal that the AMD test yields two performance measures, PAS and mean threshold, which are differentially sensitive to changes in proprioceptive acuity due to aging and neuromotor injury consequent to stroke.

## Discussion

The primary goal of this work was to develop a valid and reliable, quantitative measure of proprioceptive integrity that could be completed within a relatively short amount of time compared to currently proposed robotic tests (cf., [54, 55]). A small cohort of hemiparetic stroke survivors and a set of neurologically-intact control participants spanning a wide range of ages participated in a set of experiments that sought to evaluate the validity and reliability of the AMD test, which is a quick, ratio-scaled measure of proprioceptive integrity in the upper extremity. We successfully demonstrated that the AMD test requires just over 12 min on average to complete. The AMD test was found to be reliable across multiple testing sessions and across arms in neurologically intact control participants. We found that the AMD test provides a sensitive, quantitative assessment of proprioceptive integrity (the proprioceptive acuity score, PAS) that demonstrated good concurrent validity with a standard clinical test when discriminating between participants with intact versus clinically impaired proprioception. Moreover, the PAS has the ability to detect subtle reductions in proprioceptive acuity in some proprioceptively “intact” stroke survivors. Finally, the AMD test provides another quantitative measure (mean detection threshold, *TH*) that is able to detect subtle reductions in proprioceptive acuity in older control participants. This new assessment approach improves on previously reported methods, (e.g., [55]) in that testing can be performed fairly quickly compared to other mechanical devices (under 15 min) while still providing a ratio-scaled performance measure that is sensitive to subtle differences in proprioceptive acuity.

Applying controlled forces to the hand is a safe, effective method for testing the ability to sense arm movement. Proprioception is a complex sensation involving many different physiological sensors that transduce coupled physical parameters related to motion. These physiological sensors are sensitive to a number of different mechanical stimuli, including limb position (orientation), displacement, rate of displacement, and/or force. In developing the AMD test, we chose to apply small force perturbations to the hand rather than to impose controlled hand displacements for two reasons. First, as human limbs are inherently compliant, larger hand forces generally lead to larger hand displacements. This is true even for hemiparetic limbs that present with substantial spasticity. Indeed, we found that across all participants, the relationship between the size of the force perturbations and the magnitude of resulting hand motion was significant, with 54% of the variance explained. Second, limb spasticity could conceivably give rise to large interface forces in response to robotically-imposed hand displacements. We therefore reasoned that it would be safer to use small force perturbations in a test that may have future clinical utility. As the magnitude of force perturbations and resulting hand displacements are small, the risk of injury due to interaction with the robot is correspondingly small.

One benefit of the AMD test is that it yields a measure of proprioception for which neurologically intact participants do not automatically obtain an “ideal” score (i.e., neither a ceiling nor floor effect). When current clinical evaluations are used to test proprioception, parametric comparisons between abnormal and control groups are impossible because all the members of the control group easily obtain the highest score. In such situations, the inherent distribution of performance within the control population cannot be determined and compared to that of the clinical population. In the AMD test however, the NIC cohort yielded a range of scores that we used to define normative distributions of the *TH* and *V* dependent variables. We used these measures of central tendency and variability to define the proprioceptive acuity score (PAS), which provides a holistic measure of limb motion perception. This combined measure was sensitive to differences between hemiparetic stroke survivors with and without clinically-determined proprioceptive impairment. Even though two participants in the HSS-P group had mean *TH* scores within the range demonstrated by HSS+P participants, their *V* scores revealed the lack of true convergence to a clearly defined threshold (as demonstrated by the NIC and HSS+P participants; see Fig. 3). The PAS was sensitive to this particular performance deficit, which would have been missed if we had only assessed performance using mean detection threshold. Thus, the PAS is an appropriate measure of proprioceptive integrity after stroke because it is sensitive to changes in both the mean detection threshold and the variability of threshold assessments that we commonly observed in this population.

By contrast, the strong effect of aging on proprioceptive perception was only revealed when we considered mean motion detection threshold. This age-related effect was masked when we also included the measure of assessment variability (that is, when evaluating the correlation between age and PAS). This observation was perhaps to be expected, as the changes in proprioceptive acuity that arise due to aging in the control population almost certainly have different etiologies from those that arise in clinical populations such as the stroke survivors we studied here. For example, it is possible - albeit speculative - that the increase in detection threshold observed in the older participants in our study could be due to an age-related decrease in both the number of extrafusal muscle fibers and muscle spindles [2, 8, 64]; peripheral change in neuromuscular physiology is not likely to be the performance-limiting factor in survivors of stroke who have sensorimotor deficits arising from cortical and/or subcortical injuries.

## Conclusions

The results of this study indicate that the Arm Movement Detection (AMD) test is a quick, sensitive, and repeatable method for quantifying proprioceptive acuity. The test required about twelve minutes to complete. The resulting Proprioceptive Acuity Score - derived from the mean movement threshold (*TH*) and the variability (*V*) of 10 threshold assessments - was repeatable and able to discriminate between NIC participants and a group of participants with clinically measurable proprioceptive deficits. In the future, it may be possible to reduce test time even further. One way to do so would be to end trials when participants are confident they are at threshold instead of continuing for the full 60 s.

A limitation of the current study is that it only involves a small cohort of stroke survivors. Future studies should be conducted with a more expansive sampling of survivors with a broader range of impairment and disability to determine the extent to which changes in PAS score predict clinically meaningful deficits in sensorimotor function, as quantified using standard clinical assessments such as the Wolf Motor Function Test and/or the Chedoke-McMaster Arm and Hand Activity Inventory. The quantitative assessment of limb proprioception provided by a quick robotic assessment like the AMD test may prove to be critically important for understanding the fundamental deficits in motor coordination that are commonly present after stroke. Another potential limitation of our study was the inclusion of recipients of botulinum neurotoxin injections - a common intervention targeting spasticity. Whereas some investigators report that botulinum neurotoxin injections can increase the magnitude and stability of cortical somatosensory evoked potentials [25, 45], others report no measureable effect [6]. Using the AMD test of proprioceptive perception paired with tests of sensorimotor control in patients with and without botulinum neurotoxin injections, it may be possible to determine the extent to which changes in somatosensory evoked potentials after that therapy may reflect beneficial changes in proprioceptive sensation for perception and sensorimotor control.

A third future application of the AMD test could be to examine the natural progression of recovery of proprioceptive acuity in the first days and weeks after stroke in order to better understand the impact of sensory deficits on the recovery of functional movement and the impact of therapeutic intervention. Finally, the AMD test of proprioceptive sensation might be useful in future explorations of the benefits of therapeutic interventions based on the phenomenon of stochastic resonance on sensorimotor control after stroke. For example, Conrad and colleagues have found that performance of reaching and stabilizing actions performed with the hemiparetic arm after stroke is enhanced by applying vibrotactile “noise” to the distal arm (cf. [14–16]). Possible mechanisms of action include improved regulation of reflex excitability at the level of the spinal cord or through the augmentation of sensory input to the sensory and motor cortices, thus enhancing descending cortical control [14]. We propose that the AMD test could be used to explore mechanisms of improved sensorimotor control under vibrotactile stimulation, by assessing the extent of correlation between changes in AMD performance as a function of distal vibrotactile stimulation magnitude and corresponding changes in sensorimotor performance during reaching and stabilizing actions.

## Additional file

Additional file 1: Average AMDT thresholds and Z-scores; all data for all subjects. (XLSX 17 kb)

## Abbreviations

AMD: Arm Movement Detection
ANOVA: Analysis of variance
HSS+P: Hemiparetic stroke survivor with intact proprioception
HSS-P: Hemiparetic stroke survivor with impaired or absent proprioception
MAS: Modified ashworth scale
NIC: Neurologically intact control participants
PAS: Proprioceptive Acuity Score
*V*: A measure of detection threshold variability
*TH*: A measure of mean arm movement detection threshold
UEFM: Upper Extremity Fugl-Meyer test
